# A study of Filipina migrant workers’ subjective health in Hong Kong and an assessment of eight scoring methods for the 12-Item Short Form Health Survey (SF-12)

**DOI:** 10.3389/fsoc.2024.1420017

**Published:** 2025-01-15

**Authors:** Tim F. Liao, Rebecca Yiqing Gan

**Affiliations:** ^1^Department of Sociology, University of Illinois, Urbana, IL, United States; ^2^Fudan Institute for Advanced Study in Social Sciences, Fudan University, Shanghai, China

**Keywords:** Filipina migrant worker, SF-12, subjective mental health, subjective physical health, latent variable model, summary index, mental component summary, physical component summary

## Abstract

**Objectives:**

The SF-12 version 2 is a survey instrument for collecting data on subjective health. The US-based scoring method is the recommended standard for measuring subjective health with data collected with this instrument. The inadequacy of the US-based scoring method of the SF-12 version 2 instrument for non-US populations is widely documented. However, few studies systematically assessed relative performance of alternative scoring methods against the US-based method, our main objective in this paper. Through this investigation, we also intend to shed light on Filipina migrant workers’ subjective health in Hong Kong, our case study.

**Methods:**

This study investigates the feasibility of eight such scoring methods—six latent-variable models, the raw score index, and the US-based method—for analyzing an SF-12 version 2 instrument via a range of bootstrapped samples of varying sizes and an empirical study of the original 2017 Hong Kong Domestic Workers survey data with a set of covariates associated with Filipina migrant domestic workers’ subjective mental and physical health in Hong Kong.

**Findings:**

Our analyses favor the latent-variable factor model with the normal distribution and the identity link for analyzing the SF-12 version 2 type of data. Our empirical study of the survey data provides evidence for the beneficial effects of education, social support, and positive working conditions on migrant domestic workers’ subjective physical health and especially subjective mental health, with these two types of health analyzed *jointly* on the same measurement scale.

**Conclusion:**

For studying non-US populations with the SF-12 version 2 instrument, we recommend using the latent confirmatory factor analysis model that assumes a normal distribution and an identity link function for analyzing the MCS and PCS dimensions simultaneously.

## Introduction

The SF-12 (formally the 12-item Short-Form Health Survey) version 2 is a survey instrument for collecting data on subjective health. The US-based scoring method is the recommended standard for measuring subjective health with data collected using this instrument. The primary objective of this paper is to investigate the appropriateness of eight methods including the US-based scoring method for measuring subjective health with data collected with the SF-12 version 2 instrument. To achieve the objectives, we analyze the 2017 Hong Kong Survey of Migrant Domestic Workers by focusing on the 12-Item Short Form Health Survey (SF-12) version 2 instrument in two separate analyses: one using the original observed sample and the other, bootstrapped samples of different sizes. The SF-12 version 2 is a self-rated health questionnaire with a Mental Component Summary (MCS) and a Physical Component Summary (PCS), a survey instrument that has been widely applied in many countries. However, since the turn of the new century, studies have increasingly found that the US-based standard scoring procedure can be biased when applied to non-US settings (Hagell et al., 2017; Tucker et al., 2010, 2013, 2016; Wilson et al., 2000, 2002). Due to the problems identified, researchers began to apply country-specific scoring analyses (Tang et al., 2020; Tucker et al., 2010) although some empirical research confirmed the efficacy of the US-based scoring method for data from a nonwestern country (Younsi, 2015). The current paper analyzing Filipina migrant workers’ MCS and PCS follows up on this line of research of considering country-specific ways of scoring the SF-12 instrument.

One feasible alternative to the US-based standard procedure is to use a latent-variable confirmatory factor analytic (CFA) model for estimating MCS and PCS scores, which are unobserved and latent (Tucker et al., 2010; Younsi, 2015). However, no research so far has evaluated the comparative performance of a range of scoring methods based on the latent CFA model as well as the US-based standard scoring method. We here refer to the US-based standard scoring method by the procedure described in its application manual (Ware et al., 2002). The problem or gap in the current literature on the topic is that, to this day, there has not been a definitive study evaluating the adequacy of a whole range of competing scoring methods for analyzing data collected with the SF-12 instrument. This study aims to fill the gap.

To achieve the objective of the paper, we set out to evaluate the relative performance of eight alternative scoring methods: six estimation methods based on CFA models, the US-based standard scoring procedure, and a simple method using basic summary index scores (by averaging item raw scores). We intend to answer two questions: (1) Which of the eight scoring methods is more appropriate for analyzing data from the SF-12 instrument? (2) Using an appropriate scoring method, what can we learn about Filipina migrant workers’ subjective health in Hong Kong? We evaluate the MCS and PCS dimensions of the empirical data of the Filipina migrant sample using these eight scoring methods first before conducting a bootstrapped analysis where we compare and contrast the performances of these scoring methods by assessing how well the eight methods perform with sample size variations and with the estimation meaningfulness of some common explanatory variables taken into account.

Therefore, through the current study, this paper aims to make two significant contributions to the literature—(1) a first attempt at evaluating the relative performance of eight scoring methods with both a range of bootstrapped data and the original empirical data based on the Filipina migrant domestic workers surveyed in Hong Kong in 2017 and (2) a *joint* analysis of these female migrant workers’ subjective mental and subjective physical health when the MCS-PCS association is taken into account together, a type of analysis absent from the literature. The joint analysis also enables direct comparability of estimated MCS and PCS effects because now the two dimensions are measured on the same scale.

In the following pages, we first review the literature on migrant workers’, notably that on Filipina migrant domestic workers’ subjective health. We then analyze the 2017 Hong Kong Survey data of Filipina Migrant Domestic Workers using the eight scoring methods. Next, we introduce our analytic methods that assess the performance of the eight methods regarding sample size and substantive sensibleness and report the bootstrapped results. In our discussion section, we return to a further assessment of the empirical results from the analysis of the Filipina migrant workers, based on the insights from our bootstrapped analysis. The knowledge based on the bootstrapped analysis helps confirm which of the eight methods of scoring the SF-12 version 2 instrument can be most appropriate.

## Filipina migrant domestic workers’ subjective health

In 2023, the Philippine government reported that there were 2.16 million overseas Filipino workers (OFWs) worldwide, with women comprising a large share—57.8% (Philippine Statistics Authority, 2024). Although Sayres (2005) provided somewhat outdated data indicating that about one-quarter of Filipina workers overseas annually entered the domestic service sector, the most recent data from 2023 reflect a similar trend. Among female OFWs, more than half were engaged in elementary occupations, which obviously include domestic workers. In the same year, 77.4% of OFWs were distributed across Asian countries, with the Middle East being the primary destination. Although Hong Kong accounts for a smaller proportion, it remains an important destination for OFWs, particularly for women seeking domestic worker positions (Sayres, 2005).

Much prior research on Filipina migrant domestic workers focused on the policy and legal issues of their employment, the impact on their transnational families and children left behind, as well as the gender, racial, and class discrimination encountered (Cheng, 1996; Lan, 2006; Lee et al., 2018; Paul, 2015), with relatively few studies focused on the health aspect of such workers in the literature, despite some clear evidence that they are particularly vulnerable to adverse working conditions, material deprivation, exploitations, social isolation, and other similarly negative situations (Malhotra et al., 2013).

Adverse working conditions are harmful to these workers’ health. Excessive working hours, usually more than 13 hours per day, is a common situation that domestic workers are faced with Sum (2019). Domestic workers may also suffer from denials of rest or vacation days (Wong, 2010) and from work stress incurred by the heavy burden of caring for babies and the elderly, especially those with special needs (Lin et al., 2012). Domestic workers in Hong Kong are required to live in their employers’ homes, making their working environment also their living environment (Lai and Fong, 2020). News reports often highlight that Filipina domestic workers in certain Hong Kong households endure substandard living conditions, including improper sleeping spaces and a lack of privacy (Hollingsworth, 2017). Such poor living conditions can also be unfavorable for their health (Huang and Yeoh, 2007).

In addition to adverse working and living conditions, material deprivation may exert a negative effect on domestic workers’ health. Common types of material deprivation include wage insecurity (Wong, 2010), remittance needs of family members back home (Hall et al., 2019), exorbitant charges levied by placement agencies (Sayres, 2005), and even food deprivation (among Cambodian migrant workers; Human Rights Watch, 2005).

Moreover, domestic workers are vulnerable to various forms of abuse perpetrated by their employers. Physical assaults, verbal abuse, and sexual harassment have already been documented by several studies (Cheng, 1996; Huang and Yeoh, 2007; Ullah, 2015; Wong, 2010). Remarkably, research indicates that only a few abuse victims chose to report their cases to the police or other authorities, and such nondisclosed abuse causes additional damage to the mental health of domestic workers (Cheung et al., 2019).

Concerns for families left behind also represent one of the major constraints on domestic worker’s health. Familial connections back home can indeed offer emotional sustenance. However, these long-distance kinship network ties are oftentimes fraught with infidelity, parenting difficulty, misuse of remittances, and family misconceptions of domestic workers’ situation abroad (Hall et al., 2019). Consequently, migrant workers’ health can be negatively affected by poor-quality ties with those back in their home country. On the other hand, social network support in the host society may help relieve work stress and improve health conditions. However, social isolation is prevalent among domestic workers. Language barriers, cultural divides, and explicit or implicit discrimination by local residents impede their integration (Asian Migrants Centre, 2001; Ward et al., 1999). Some domestic workers are even faced with mobility and social restrictions. They are restricted from independent outings, maintaining online communication with their families, and regular interaction with domestic worker peers (International Organization for Migration, 2003). Despite these challenges, engaging in religious practices during their off-hours can help Filipina domestic workers build social ties and reduce their emotional cost of working overseas (Nakonz and Shik, 2009).

A final potentially important factor related to Filipina domestic workers’ health is migration trajectory. Many domestic workers migrated to more than one place, with some of them following a stepwise migration pattern to work their way up the destination hierarchy (Paul, 2011, 2017) and others embedded in a precarity chain of transnational labor migration (Parreñas et al., 2018). Previous studies have demonstrated that the complexity of migration trajectories is associated with migrant workers’ job satisfaction (Liao and Gan, 2020), which in turn can significantly impact their mental well-being.

Due to the outlined risk factors, domestic workers frequently experience health issues. They commonly report work-related physical health issues, such as back and joint pain, allergic reactions, and musculoskeletal strains (Hanley et al., 2011; Labao, 2021). On the mental health side, a considerable proportion of them suffer from psychotic, neurotic, and mood disorders (Holroyd et al., 2001; Zahid et al., 2002). The studies previously mentioned have highlighted the diverse causes of health challenges among Filipina domestic workers. However, there is a notable lack of research focused on systematically and scientifically measuring their subjective mental and physical health at the same time and identifying underlying reasons.

A few exceptions stand out in the scientific measurement of health among Filipina domestic workers in Hong Kong. One is a study by Bagley et al. (1997), which used a formal scale to assess mental health; however, this study is nearly 30 years old, had a small sample size, and focused solely on mental health. A more recent study by Sumerlin et al. (2024) also used a scientific scale to measure mental health but focused only on specific symptoms, namely depression and anxiety. Both studies overlooked physical health in their assessments. The other exception is the research conducted by Chung and Mak (2020), which used the same dataset as the current study. However, their study did not critically evaluate the appropriateness of the subjective health scoring method and did not examine physical and mental health by integrating them into the same model despite the well-established significant correlation between physical and mental health.

In summary, the extant literature has not adequately addressed two key issues: (1) how to establish a scientific method for measuring the overall health status of this specific migrant worker group, moving beyond focusing on isolated health symptoms; and (2) how to develop a systematic approach to examining the factors influencing health status of this migrant group, particularly by jointly considering mental and physical health. In this context, the current study represents a first effort to bridge these two gaps in the research literature.

In a later section, we will report the estimated associations between many of the factors cited above and the subjective mental and physical health of a large sample of Filipina migrant domestic workers in Hong Kong surveyed in 2017, using the SF-12 (version 2) scale via eight different scoring methods.

## Methods

### The instrument

The SF-12 version 2 consists of 12 questions spanning eight health domains: Physical Functioning (PF), Role Physical (RP), Bodily Pain (BP), General Health (GH), Vitality (VT), Social Functioning (SF), Role Emotional (RE), and Mental Health (MH) for surveying one’s physical and mental health. For a detailed view of the items and the response categories in each domain, please see Table 1. It is clear from the table that the responses may have either three or five categories, and all are ordered along an ordinal scale.

**Table 1:** The SF-12 version 2 survey instrument.

Domain	Items	Responses
Physical functioning (PF)	Moderate activities, such as moving a table, pushing a vacuum cleaner, bowling, or doing tai chi	For both items: “Yes, limited a lot,” “Yes, limited a little,” “No, not limited at all.”
	Climbing several flights of stairs	
Role physical (RP)	Accomplished less than you would like as a result of your physical health	For both items: “All of the time,” “Most of the time,” “Some of the time,” “A little of the time,” “None of the time.”
	Were limited in the kind of work or other activities as a result of your physical health	
Bodily pain (BP)	How much did pain interfere with your normal work (including both work outside the home and housework)	“Not at all,” “A little bit,” “Moderately,” “Quite a bit,” “Extremely.”
General health (GH)	In general, would you say your health is	“Excellent,” “Very good,” “Good,” “Fair,” “Poor.”
Vitality (VT)	Did you have a lot of energy	“All of the time,” “Most of the time,” “Some of the time,” “A little of the time,” “None of the time.”
Social functioning (SF)	How much of the time has your physical health or emotional problems interfered with your social activities (like visiting friends, relatives, etc.)	“All of the time,” “Most of the time,” “Some of the time,” “A little of the time,” “None of the time.”
Role emotional (RE)	Accomplished less than you would like as a result of any emotional problems	For both items: “All of the time,” “Most of the time,” “Some of the time,” “A little of the time,” “None of the time.”
	Did work or other activities less carefully than usual as a result of any emotional problems	
Mental health (MH)	Have you felt calm and peaceful	For both items: “All of the time,” “Most of the time,” “Some of the time,” “A little of the time,” “None of the time.”
	Have you felt downhearted and depressed	

The SF-12 instrument is most often employed with the US-based scoring procedure. The procedure is referred to as the US-based scoring method or standard because the means and standard deviations used in the standardization process as well as the factor score coefficients used in aggregation are derived from the 1998 general U.S. population. See Method 1 in Appendix A and Ware et al. (2002) for further technical details.

### The design

To properly assess eight different scoring methods for analyzing data from the SF-12 instrument for measuring subjective health, we employ two approaches to analyzing the empirical data: an analysis of the original empirical survey data and an analysis of bootstrapped survey data of a range of sample sizes to gain a better understanding of the performance of the eight scoring methods.

### The basic CFA model

Previous research reported the application of a confirmatory factor analysis (CFA) on the SF-12 instrument (or its uncondensed 36-item longer version SF-36) for obtaining the scoring coefficients for computing factor scores (Tucker et al., 2010; Younsi, 2015). Such an application has the option of using a CFA model with ordinal observed indicators. Along these lines, we view PCS and MCS as two latent (i.e., unobserved) variables, *x*_1*i*_ and *x*_2*i*_, with the observed 12 items for estimating the latent variables for individual *i*. Our CFA model is a special case of the generalized structural equation model. Just like any generalized linear models, a variety of link functions can be used. For the six observed MCS items *y*_1*ij*_ for *j* = 1 to 6 and case *i* = 1 to *N*, we have:


(1) $$g\left(y_{1ij}\right)=\alpha_{jk}+\beta_{j}x_{1i}+\varepsilon_{1ij}$$

where *α_jk_* represents the *k*th threshold value between adjacent pairs of the observed five ordered categories in the *j*th item, *β_j_* is the parameter linking the latent MCS unobserved variable *x*_1*i*_ to the *j*th item *y*_1*ij*_, ε _1*ij*_ is the random measurement error for the *j*th item *y*_1*ij*_, and g(·) is the link function. In this case, an ordinal logit link is a candidate because of the ordered nature of the observed categories. There are six observed indicators *y*_1*ij*_ for *j* = 1 to *J* or 1 to 6, each of which has five (or three) ordered response categories. The PCS dimension represented by *y*_2*ij*_ is expressed similarly except that two of the six items have three ordered response categories instead of five, as is the case of the SF-12 version 2 instrument (see Table 1). Our theoretical MCS and PCS structure follows that set out by Ware and colleagues and verified by Tucker and colleagues’ estimation with the addition of the correlation between the latent MCS and PCS dimensions (Tucker et al., 2016; Ware et al., 1995). This way, we can estimate the latent scores of both *y*_1*ij*_ and *y*_2*ij*_ simultaneously in a CFA model with the two correlated factors (*y*_1*ij*_ and *y*_2*ij*_).

### Further CFA modeling developments

The latent CFA approach has at least three major advantages over the standard US-based scoring procedure. First, the strength of the relation between the SF-12 items and MCS/PCS is estimated directly from the data. Second, no assumption is made about the distances between any of the adjacent ordered categories, which are estimated with the *α_jk_* parameters. A third advantage is related to yet different from the second—people from different cultures or origins may have different thresholds (such as bodily pain thresholds) and do not have identical interpretation of ordered categories such as “all the time,” “most of the time,” “some of the time,” “a little of the time,” and “none of the time”; or more generally, the assumption of the same set of thresholds that separate any ordinal responses across different groups of people may not hold, especially across people of different societies or cultures (King et al., 2003; Ware et al., 1995). Similarly, for the SF-12 instrument, we have ordinal categories indicating a latent mental or physical health dimension. The flexibility to estimate without requiring a fixed set of thresholds provides cultural specificity instead of using a one-size-fits-all procedure which enforces identical thresholds.

### Analytic strategy for the Filipina sample

To fully engage the literature on migrant Filipina domestic workers, we analyzed the data for the Filipina migrant workers from the 2017 Hong Kong Survey of Female Filipino and Indonesian Migrant Domestic Workers, a multistage random sample based on face-to-face interviews of about 2,000 such workers (Chung et al., 2020), by including all relevant factors available from the survey data that were discussed in the literature (Asian Migrants Centre, 2001; Cheng, 1996; Cheung et al., 2019; Chung and Mak, 2020; Hall et al., 2019; Hanley et al., 2011; Hollingsworth, 2017; Holroyd et al., 2001; Huang and Yeoh, 2007; Human Rights Watch, 2005; International Organization for Migration, 2003; Labao, 2021; Lan, 2006; Lee et al., 2018; Liao and Gan, 2020; Lin et al., 2012; Malhotra et al., 2013; Nakonz and Shik, 2009; Parreñas et al., 2018; Paul, 2011, 2015, 2017; Sayres, 2005; Sum, 2019; Ullah, 2015; Ward et al., 1999; Wong, 2010; Zahid et al., 2002).

Our seemingly unrelated regression (SURE) analysis includes both the MCS and the PCS dimensions in the same models because such analysis extends the typical linear regression by allowing unobserved correlated random errors between a MCS sub-model and a PCS sub-model—that is, the correlation between a part of *y*_1*ij*_ and a part of *y*_2*ij*_ unrelated to the explanatory variables in each of the two regressions. This method allows us to estimate structural effects such as the effects of background and employment factors on subjective health by modeling mental and physical health simultaneously because these two dimensions of subjective health are correlated through the error term, which captures the correlated portion of mental and physical health unexplained by the structural factors. This allows a clean estimation of the structural effects and it allows the two dimensions to be measured on the same scale. The MCS and PCS scores were first obtained using one of the eight methods, six of which are based on a CFA model. It is important to evaluate all eight scoring methods since there is no available scoring procedure specifically designed for the Filipino population, let alone a Filipina migrant worker population. We then included the estimated MCS and PCS scores as outcome variables in the regression analysis together with all potential factors related to migrant domestic workers’ health reviewed in an earlier section available in our survey data. The bottom half of Table 2 presents the descriptive statistics of the variables (including their definitions) used in the analysis of the Filipina migrant workers’ MCS and PCS.

**Table 2:** Descriptive statistics of the Filipina sample.

	Mean	Std. Dev.	Min	Max
Subjective health conditions				
Mental component score (MCS)				
Method 1	44.47	8.38	21.79	73.07
Method 2	3.48	0.61	1.67	5
Method 3	−0.08	1.03	−2.77	2.39
Method 4	−0.03	0.43	−1.10	0.76
Method 5	−0.08	1.03	−2.78	2.43
Method 6	−0.03	0.43	−1.11	0.77
Method 7	−0.03	0.49	−1.43	1.04
Method 8	Not concave			
Physical component score (PCS)				
Method 1	46.40	7.31	21.81	66.37
Method 2	3.61	0.68	1.89	5
Method 3	−0.06	0.59	−1.65	1.29
Method 4	−0.02	0.20	−0.48	0.33
Method 5	−0.01	0.11	−0.31	0.23
Method 6	−0.00	0.05	−0.12	0.08
Method 7	−0.02	0.28	−0.85	0.60
Method 8	Not concave			
Sociodemographic characteristics				
Age (in years)	35.43	7.64	20	74
Education				
Primary school and below (reference)	2.00			
Secondary or vocational	49.00			
Post-secondary (post-secondary technical school/university graduate/postgraduate degrees)	49.00			
Have partner (1 = yes)	0.63	0.48	0	1
Have child (1 = yes)	0.67	0.47	0	1
Speak English well (1 = yes)	0.94	0.24	0	1
Speak Cantonese well (1 = yes)	0.06	0.23	0	1
Financial burden				
Remittance (ordered categories by frequency of sending remittance home)	2.97	0.25	1	4
Agency fee (1 = needed to pay)	0.67	0.47	0	1
Social support				
Friendship ties in HK (ordered categories by frequency of contact)	3.50	0.62	0	4
Friendship ties in home country (ordered categories by frequency of contact)	2.68	0.96	0	4
Participation of religious activities (1 = yes)	0.63	0.48	0	1
Working conditions				
Have a private room (1 = yes)	0.56	0.50	0	1
Working hours (per day)	13.84	2.46	7	24
Monthly income (in HK$1,000)	4.28	0.21	3.21	7
Have bonuses/gifts (1 = yes)	0.76	0.43	0	1
Back pay experience (1 = yes)	0.04	0.21	0	1
Employer’s attitudes (larger number indicates poorer attitude)	0.83	1.45	0	9
Migration trajectory				
Entropy (complexity of past migration history in the [0, 1] range)	0.18	0.13	0.01	0.59
*N*	1,098			

We estimated altogether eight SURE models in the analysis. Model 1 (based on Method 1) includes the MCS and PCS measures based on the standard US-based scoring method (Ware et al., 2002). Model 2 is based on the summed measures of the raw scores of the MCS and PCS sub-dimensions, respectively. Model 3 formalizes Equation 1 with an ordinal logit link function. Model 4 is similar to Model 3 except with an identity link based on a Gaussian (normal) distribution assumption, which is one of the models tested in a previous study (Chum et al., 2016). Models 5 and 6 are modified versions Models 3 and 4 by allowing cross-loaded General Health (GH) and Vitality (VT) subscales as applied in a prior study (Kathe et al., 2018). Model 7 extends Model 3 by allowing for correlated errors with paired subscale items as done by previous research (Chum et al., 2016; Lau et al., 2021). Finally, Model 8 combines Models 6 and 7 by allowing for both cross-loaded GH and VT subscales as well as correlated errors between paired subscale items. As shown in Appendix A (Methods 5, 6, and 8), “cross-load” refers to the practice of allowing both the General Health (GH) and Vitality (VT) items to contribute to the scoring of both PCS and MCS. This approach was applied in a prior study by Kathe et al. (2018). We did not consider models with an ordinal logit link function and correlated errors between paired items because such models are not found in the literature and because our preliminary analysis showed convergence issues with such models. For a formal specification of the eight scoring methods for the estimation of the eight models used in the analysis, see Appendix A.

### Analytic strategy for assessing sample size effects

We designed an analysis via bootstrapping for assessing the performances of the six latent variable methods vis-à-vis those of the standard scoring procedure and of the summary index method using raw scores. The rationale for such a study of the SF-12 lies in the unobserved nature of subjective health. For studying objective health, one can rely on simulations because simulated data can be based on some observed values of BMI or blood pressure, for example.

In the study, we examine an aspect of the performance of the eight scoring methods by randomly drawing 500 samples with replacement from the survey data of the female Filipina migrant workers in Hong Kong described above. The purpose of this analysis is to employ our empirically observed data (instead of creating data hypothetically without sufficient empirical foundation) by estimating the correlated structures of mental and physical health as part of a seemingly unrelated regression model and see how computationally feasible the eight scoring methods are and how sensible the estimates can be from a substantive point of view. In other words, we estimated the MCS and PCS models with two regressions by assuming these two regression models are correlated, hence using seemingly unrelated regressions.

We designed the bootstrapped analysis in such a way that we would be able to see the effect of sample size by varying the randomly drawn 500 samples from the Filipina data with replacement in four sample sizes, 300, 600, 900, and 1,200. The purpose of using varying sample sizes is to see if a particular method can be more sensitive to sample size variations, especially when the size is small, and if the distribution range of a certain estimate can be particularly large. This analysis has another purpose—to see whether a given method may yield senseless estimates, based on what we know from the literature. For example, higher education is typically positively associated with better health outcomes, both in the mental and in the physical dimension. If a method yields estimates contrary to the literature, then it is a strong indication that this particular method may not be desirable to use. For practical purposes later when we discuss results, we will focus on only the MCS results because estimates in the MCS and PCS are consistently correlated.

## Results

### Results from the initial analysis of the Filipina data

Table 3 reports the estimated results from the seven SURE regressions defined above. We set out to estimate the regressions with two objectives—a statistical assessment of the efficacy of the eight different scoring methods of the MCS and PCS dimensions and a substantive understanding of the factors associated with Filipina migrant domestic workers’ mental and physical health in Hong Kong. Statistical efficacy refers to two qualities here: (1) The estimated MCS-PCS correlation ideally falls with a reasonable positive range (yet without reaching unity), and the etimates of covariates are consistent with the literature; (2) a method based on a given model can converge regardless of sample size. We deal with our first objective below first. Once we have established that one or more models/methods are more appropriate than the others, we will proceed with the second objective.

**Table 3:** Seemingly unrelated regression models for estimating the association between mental health and physical health of Filipina domestic workers.

	Method 1		Method 2		Method 3		Method 4		Method 5		Method 6		Method 7	
	MCS	PCS	MCS	PCS	MCS	PCS	MCS	PCS	MCS	PCS	MCS	PCS	MCS	PCS
Correlation of residuals	−0.184		0.471		0.685		0.641		0.685		0.641		0.991	
Sociodemographic characteristics														
Age	0.096** (0.035)	0.003 (0.031)	0.007** (0.002)	0.002 (0.003)	0.009* (0.004)	0.004 (0.002)	0.003+ (0.002)	0.001 (0.001)	0.010* (0.004)	0.001 (0.000)	0.003+ (0.002)	0.000 (0.000)	0.004* (0.002)	0.002+ (0.001)
Education (ref: primary school and below)														
Secondary or vocational	0.656 (1.763)	2.051 (1.562)	0.153 (0.126)	0.252+ (0.143)	0.485* (0.210)	0.227+ (0.123)	0.197* (0.088)	0.082+ (0.043)	0.481* (0.210)	0.042+ (0.023)	0.196* (0.088)	0.021+ (0.011)	0.253* (0.099)	0.149** (0.058)
Post-secondary	0.180 (1.757)	1.959 (1.557)	0.102 (0.125)	0.219 (0.143)	0.351+ (0.209)	0.190 (0.123)	0.142 (0.087)	0.070 (0.043)	0.349+ (0.210)	0.035 (0.023)	0.141 (0.088)	0.018 (0.011)	0.191+ (0.098)	0.115* (0.057)
Have partner	−1.451* (0.673)	−0.212 (0.596)	−0.113* (0.048)	−0.042 (0.055)	−0.113 (0.080)	−0.024 (0.047)	−0.044 (0.033)	−0.006 (0.016)	−0.115 (0.080)	−0.004 (0.009)	−0.045 (0.034)	−0.001 (0.004)	−0.053 (0.038)	−0.028 (0.022)
Have child	0.818 (0.718)	−0.656 (0.636)	0.037 (0.051)	−0.044 (0.058)	0.014 (0.086)	−0.017 (0.050)	0.016 (0.036)	−0.002 (0.017)	0.015 (0.086)	−0.003 (0.009)	0.017 (0.036)	−0.001 (0.004)	−0.012 (0.040)	−0.010 (0.023)
Speak English well	−0.378 (1.003)	0.918 (0.889)	0.007 (0.072)	0.131 (0.081)	0.211+ (0.120)	0.141* (0.070)	0.101* (0.050)	0.055* (0.024)	0.208+ (0.120)	0.026* (0.013)	0.099* (0.050)	0.014* (0.006)	0.120* (0.056)	0.071* (0.033)
Speak Cantonese well	−0.745 (1.051)	−1.121 (0.932)	−0.105 (0.075)	−0.117 (0.085)	−0.088 (0.125)	−0.029 (0.073)	−0.032 (0.052)	−0.007 (0.026)	−0.088 (0.125)	−0.005 (0.014)	−0.033 (0.052)	−0.002 (0.007)	−0.068 (0.059)	−0.042 (0.034)
Financial burden														
Remittance	−0.274 (0.998)	−1.411 (0.885)	−0.056 (0.071)	−0.124 (0.081)	0.045 (0.119)	−0.114 (0.070)	0.023 (0.050)	−0.039 (0.024)	0.043 (0.119)	−0.021 (0.013)	0.022 (0.050)	−0.010 (0.006)	−0.043 (0.056)	−0.034 (0.033)
Agency fee	−1.865*** (0.529)	−1.311** (0.469)	−0.177*** (0.038)	−0.164*** (0.043)	−0.226*** (0.063)	−0.110** (0.037)	−0.096*** (0.026)	−0.032* (0.013)	−0.226*** (0.063)	−0.020** (0.007)	−0.096*** (0.026)	−0.008* (0.003)	−0.133*** (0.030)	−0.077*** (0.017)
Social support														
Friendship ties in HK	0.367 (0.420)	0.741* (0.372)	0.038 (0.030)	0.078* (0.034)	0.118* (0.050)	0.060* (0.029)	0.055** (0.021)	0.023* (0.010)	0.118* (0.050)	0.011* (0.005)	0.055** (0.021)	0.006* (0.003)	0.066** (0.024)	0.039** (0.014)
Friendship ties in home country	−0.153 (0.264)	0.708** (0.234)	0.009 (0.019)	0.061** (0.021)	0.042 (0.031)	0.052** (0.018)	0.011 (0.013)	0.015* (0.006)	0.042 (0.032)	0.010** (0.003)	0.011 (0.013)	0.004* (0.002)	0.027+ (0.015)	0.018* (0.009)
Religious activities	0.366 (0.540)	−1.334** (0.479)	−0.011 (0.039)	−0.119** (0.044)	−0.253*** (0.064)	−0.153*** (0.038)	−0.109*** (0.027)	−0.054*** (0.013)	−0.251*** (0.064)	−0.029*** (0.007)	−0.108*** (0.027)	−0.014*** (0.003)	−0.127*** (0.030)	−0.073*** (0.018)
Working conditions														
Have a private room	0.898+ (0.495)	−0.347 (0.439)	0.050 (0.035)	−0.017 (0.040)	0.046 (0.059)	−0.014 (0.035)	0.017 (0.025)	−0.009 (0.012)	0.047 (0.059)	−0.003 (0.006)	0.018 (0.025)	−0.002 (0.003)	0.007 (0.028)	0.001 (0.016)
Working hours (per day)	0.109 (0.102)	−0.106 (0.090)	0.003 (0.007)	−0.009 (0.008)	−0.005 (0.012)	−0.003 (0.007)	−0.003 (0.005)	−0.001 (0.002)	−0.005 (0.012)	−0.001 (0.001)	−0.003 (0.005)	−0.000 (0.001)	−0.004 (0.006)	−0.003 (0.003)
Monthly income (1,000 HKD)	2.847* (1.169)	0.720 (1.036)	0.206* (0.083)	0.138 (0.095)	0.281* (0.139)	0.137+ (0.082)	0.098+ (0.058)	0.040 (0.028)	0.287* (0.140)	0.025+ (0.015)	0.100+ (0.058)	0.010 (0.007)	0.140* (0.066)	0.078* (0.038)
Have bonuses/gifts	2.314*** (0.585)	1.157* (0.519)	0.208*** (0.042)	0.178*** (0.048)	0.358*** (0.070)	0.163*** (0.041)	0.160*** (0.029)	0.057*** (0.014)	0.358*** (0.070)	0.030*** (0.008)	0.161*** (0.029)	0.015*** (0.004)	0.188*** (0.033)	0.107*** (0.019)
Back pay experience	−5.748*** (1.184)	−0.320 (1.049)	−0.407*** (0.084)	−0.171+ (0.096)	−0.612*** (0.141)	−0.186* (0.083)	−0.263*** (0.059)	−0.059* (0.029)	−0.616*** (0.141)	−0.034* (0.015)	−0.265*** (0.059)	−0.015* (0.007)	−0.276*** (0.066)	−0.146*** (0.039)
Employer’s attitudes	−0.309+ (0.172)	0.055 (0.152)	−0.021+ (0.012)	−0.008 (0.014)	−0.045* (0.020)	−0.004 (0.012)	−0.022* (0.009)	−0.002 (0.004)	−0.045* (0.020)	−0.001 (0.002)	−0.022* (0.009)	−0.001 (0.001)	−0.019* (0.010)	−0.009+ (0.006)
Migration trajectory														
Entropy	−0.279 (1.856)	0.611 (1.645)	−0.021 (0.132)	−0.030 (0.151)	−0.420+ (0.221)	−0.223+ (0.130)	−0.172+ (0.092)	−0.069 (0.045)	−0.413+ (0.221)	−0.042+ (0.024)	−0.168+ (0.092)	−0.018 (0.011)	−0.163 (0.104)	−0.086 (0.061)
Constant	26.965*** (6.678)	43.092*** (5.919)	2.237*** (0.477)	2.784*** (0.542)	−2.594** (0.796)	−0.952* (0.466)	−0.978** (0.332)	−0.295+ (0.163)	−2.617** (0.797)	−0.174* (0.086)	−0.982** (0.333)	−0.075+ (0.041)	−1.104** (0.374)	−0.598** (0.218)
R-squared	0.086	0.057	0.109	0.086	0.132	0.097	0.137	0.091	0.131	0.097	0.137	0.091	0.154	0.148
*N*	1,098													

Method 8’s model cannot be executed due to convergence failure during score generation on the observed data; Standard errors in parentheses; ***p < 0.001, **p < 0.01, *p < 0.05, +*p* < 0.1.

Examining the estimates across the columns, we see that there are indeed noticeable differences in both the size of the coefficient estimates and in their statistical significance across estimation methods. A place to begin is the correlated errors between the MCS and the PCS sub-models in a SURE regression because a given correlated error between two such sub-models indicates the correlation between the latent MCS and PCS dimensions not captured by the factors in the regression analysis. To put this evaluation in perspective, typically, a reasonable MCS-PCS correlation is in the moderately strong positive range (e.g., about 60%, see Farivar et al., 2007). Judged by this statistic, three methods stand out. Method 1 that relies on the standard scoring procedure gave a negative correlation while Method 7 yielded correlations close to unity. Neither of them appears to be reasonable. We will as well apply this criterion in the next subsection when we consider bootstrapped results. In the current analysis, Method 8 failed to converge for analyzing the full sample of the observed data, thus not reported in Table 3. To obtain a better assessment of the relative performance of these scoring methods especially in terms of the estimates of the covariates and in terms of sample size variation, we turn to bootstrapped analysis below.

### Results from the bootstrapped samples

**Figure 1:**
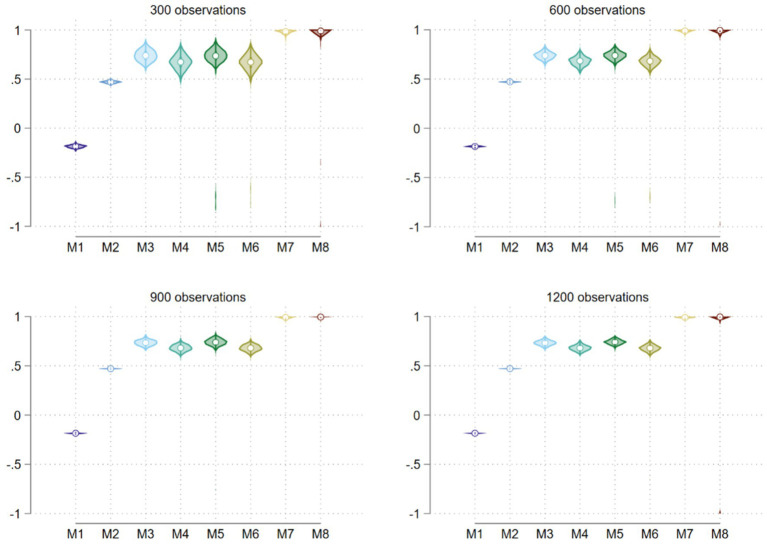
Residual correlation distributions from a bootstrapped analysis using eight methods.

We present the main results from our bootstrapped analysis in two figures. Figure 1 presents the violin plots for the residual correlation (i.e., MCS and PCS correlation) by method and sample size. It is obvious that Methods 1, 7, and 8 produced unreasonable estimated MCS and PCS correlations, with Method 1 yielding negative correlations while Methods 7 and 8, extremely high correlations close to unity regardless of sample size. None of these results are reasonable, judged by the criterion described above.

**Figure 2:**
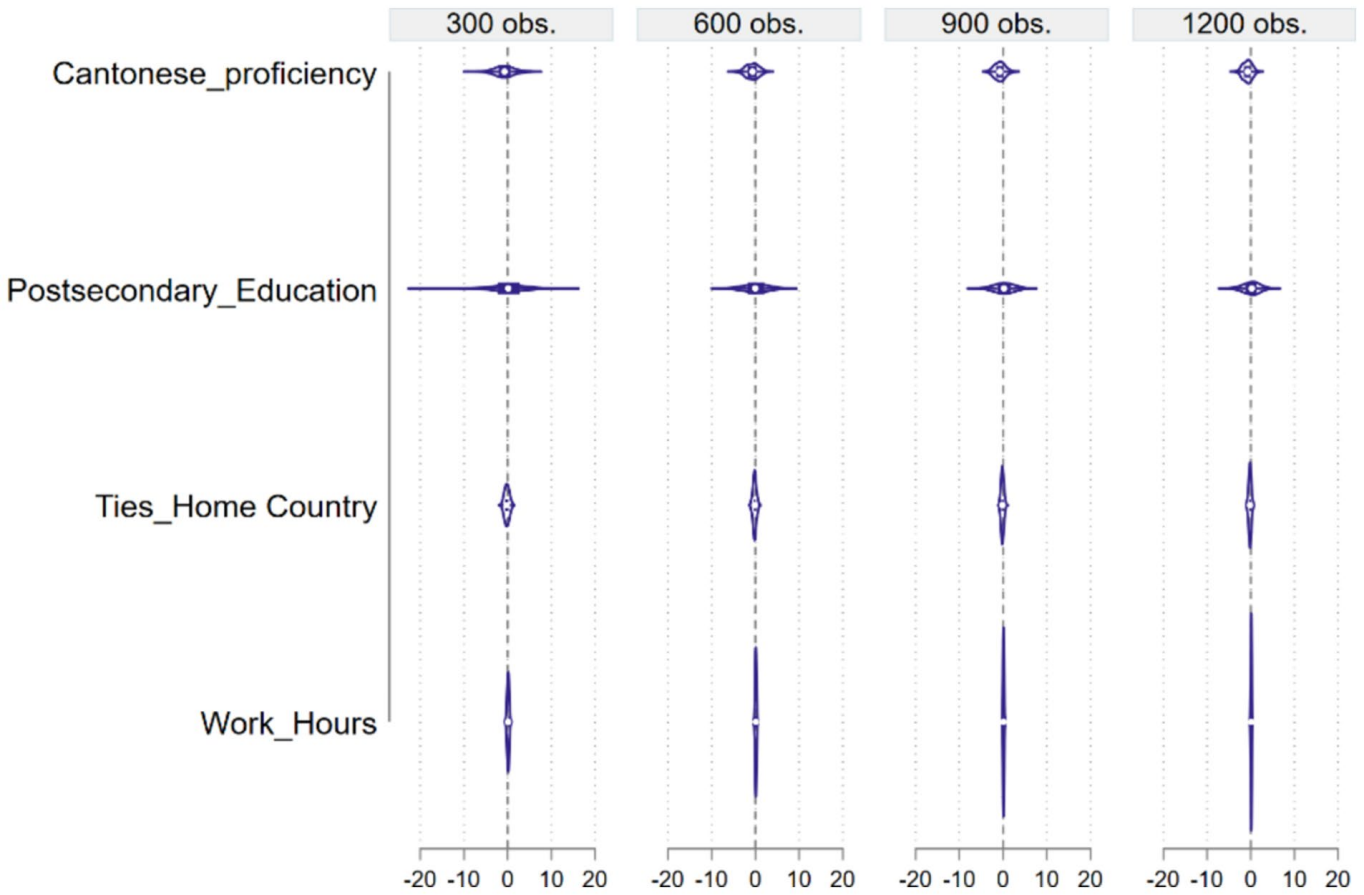
MCS estimate distributions by explanatory variable for Method 1.

**Figure 3:**
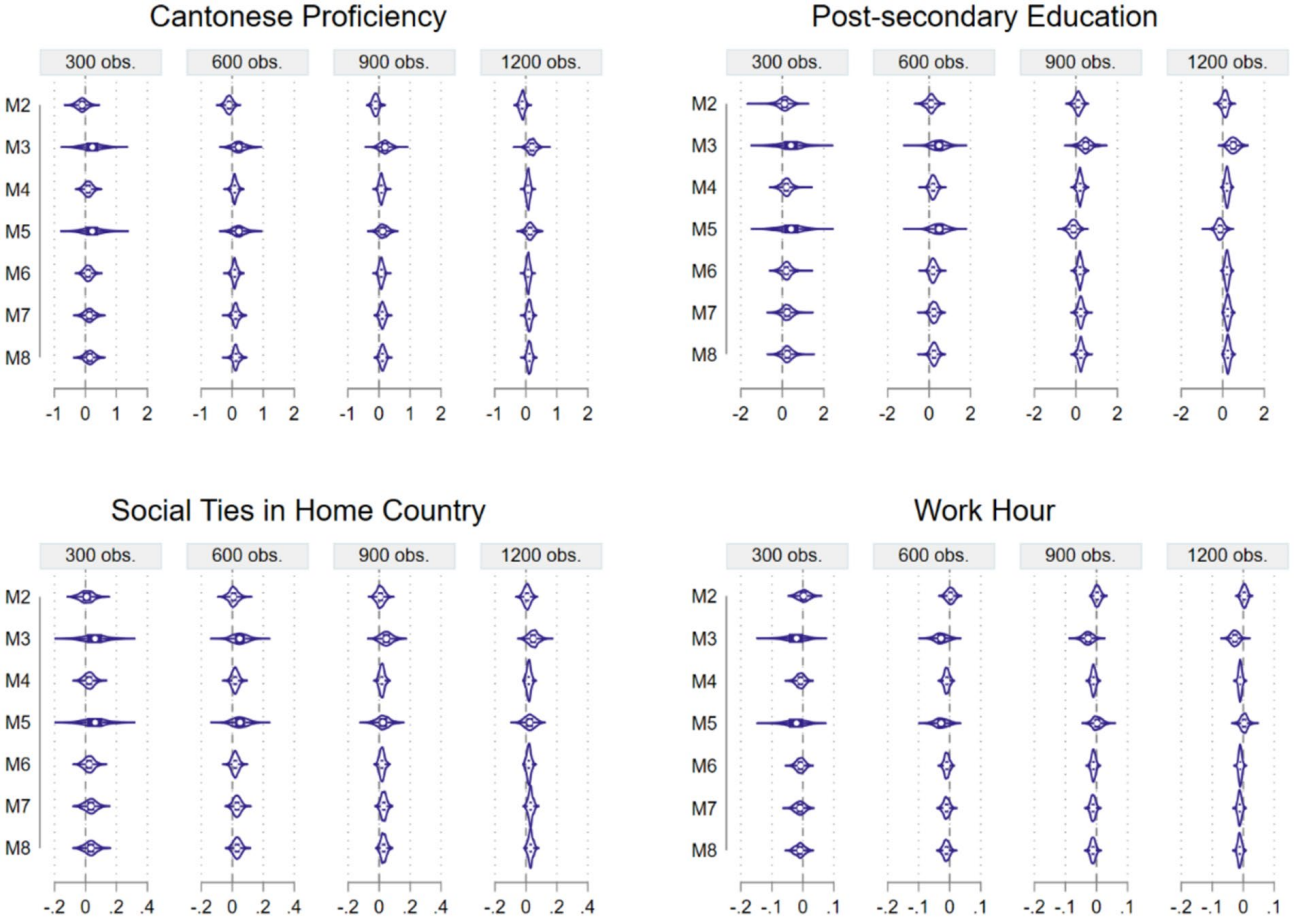
MCS Estimate distributions of four explanatory variables for Methods 2 to 8.

Because the MCS coefficient estimate distributions for Method 1 are much wider than the other methods, to facilitate easier comparisons, we separately present all estimate distributions of four sets of estimate distributions for Method 1 and for the other seven methods in Figures 2, 3 for the estimates of Cantonese proficiency, postsecondary education, network ties in home country, and work hours.

From Figure 2, we see that overall, Method 1 produced a wide range of estimate distributions for most explanatory variables, judged by the X-axis scale in the subplots although the range narrows somewhat with an increase in sample size. Let us focus on the four explanatory variables mentioned above, Cantonese proficiency, postsecondary education, network ties in home country, and work hours. According to Method 1, a Filipina’s ability to speak Cantonese well has a negative effect on her mental health, postsecondary education has no effect, ties in her home country has a slightly negative effect, and work hours has no effect, with the entire density distributions concentrating around zero. All these results are either contrary to or inconsistent with the literature. In comparison, most of the other methods yielded more meaningful results. Let us again focus on the estimate distributions of Cantonese proficiency.

Although almost all Filipina migrant workers are proficient in English, having Cantonese proficiency should boost their confidence and thus mental health because most of their employers are Cantonese speaking. Here, most methods except Method 2 produced correct estimates on average, though Methods 4, 6, 7, and 8 have narrower distribution ranges, and such ranges get narrower with sample size, as expected. A similar observation can also be made about the postsecondary education estimate distributions, also presented in Figure 3.

Here we see a similar set of comparisons across the seven methods. Method 2 did produce correct average estimates on the positive side this time because higher education improves one’s mental health. Again, Methods 4, 6, 7, and 8 have narrower distribution ranges, which narrow with sample size. Such ranges are much narrower than the distribution ranges for Method 1, judged by the X-axis scale in the figures. We continue our comparison of the methods with the next explanatory variable, social ties in home country, in Figure 3.

In this figure, we see that all methods from Methods 2 to 8 yielded positive average social network estimates as expected because having better social network connections should improve one’s mental health. Once again, Methods 4, 6, 7, and 8 are to be favored because of their narrower estimate distribution ranges. The two methods (M3 and M5) using the ordinal logit link function and assuming the logistic distribution produced a much wider estimate distribution. Finally, let us examine estimate distributions of the explanatory variable work hours (Figure 3).

When a female migrant domestic worker has extended work hours, such working conditions could add stress and be harmful to their mental health. So, we expect a negative average effect here. This is what we see for most methods except Method 2 and Method 5 (for two of the sample sizes). Once again, Methods 3 and 5 both have wider estimate distribution ranges, and Methods 4, 6, 7, and 8 outperform the other methods in having a correct average effect and a narrower estimate distribution range.

## Discussion

### Bootstrapped analysis of different sample sizes

So far, we have found good support for Methods 4, 6, 7, and 8. Methods 4 and 6 both assume a normal distribution and use an identity link function, with Method 6 allowing for the cross-loaded General Health and Vitality subscales. The two methods differ little in the average and the range of their estimates. In comparison, Methods 7 and 8 typically have narrower estimate distribution ranges, often clear of zero, especially for larger sample sizes. This suggests that all these four methods can be considered.

At this point, we would like to bring in another piece of evidence from our bootstrapped exercise—convergence complications. Methods 7 and 8, because they involve correlated measurement errors, had a more difficult time to achieve convergence during our bootstrapped analysis. We include this information in Table 4, where the number of times lacking convergence out of the 500 bootstrapped samples are presented by method and sample size.

**Table 4:** Number of times lacking convergence out of 500 by method and sample size.

	Step 1: Generating MCS and PCS				Step 2: Estimating seemingly unrelated regression models				Steps 1 and 2 together			
	300 obs.	600 obs.	900 obs.	1,200 obs.	300 obs.	600 obs.	900 obs.	1,200 obs.	300 obs.	600 obs.	900 obs.	1,200 obs.
Method 1	/	/	/	/	/	/	/	/	/	/	/	/
Method 2	/	/	/	/	/	/	/	/	/	/	/	/
Method 3	181	106	56	52	/	/	/	/	181	106	56	52
Method 4	/	/	/	/	/	/	/	/	/	/	/	/
Method 5	180	131	56	48	/	/	/	/	180	131	56	48
Method 6	/	/	/	/	/	/	/	/	/	/	/	/
Method 7	/	/	/	/	98	79	29	37	98	79	29	37
Method 8	102	63	34	20	62	45	14	9	164	108	48	29

Clearly, Methods 3, 5, 7, and 8 all experienced some difficulty with convergence, especially when sample sizes are small (smaller than 900). When they are large, say greater than 900, Method 8 appears to be a feasible choice for use in estimating the MCS and PCS dimensions. Both Methods 3 and 5 implement a logit link and Methods 7 and 8 involve correlated errors though Method 8 also includes the cross-loaded General Health and Vitality subscales.

Therefore, our recommendation based on the bootstrapped analysis is to use method 4 or 6 when sample size is small, and when sample size is larger than at least 900, Method 8 can be considered. If convergence for the method is a problem, then the researcher can return to using Method 6 (or 4).

### Another look at the analysis of the Filipina migrant workers in Hong Kong

With the knowledge gained from our bootstrapped study, let us return to the empirical analysis of the Filipina migrant domestic workers’ subjective mental and physical health reported in Table 3. The estimates from the models using Methods 4 and 6 are almost identical, without any real differences in any statistical significance tests. The same observation can be made about Methods 7, with estimates in a reasonable range. To have a concise interpretation of the results, we focus on those from Method 6, because the model-adjusted correlation between MCS and PCS from Method 7 is >0.99 while that from Method 6 is 0.642, a sizeable correlation without being unreasonable and very consistent with what was suggested in the literature (Farivar et al., 2007).

The results are largely consistent with those reported in the literature. Having a higher level of education tends to show an improved level of mental wellbeing (especially supported by the contrast of those with a secondary education against those with just a primary education). A female migrant worker having had financial burdens in the form of agency fees showed a lower level of mental and physical health (Sayres, 2005). Various forms of social support are associated with improvement in both mental and physical wellbeing. Having friendship ties in Hong Kong has a positive association with a worker’s mental and physical health although having such ties with friends back in the home country has a positive correlation with one’s physical health only, providing some positive evidence on social support effect updating the findings from prior research (Cheung et al., 2019). Additionally, the findings show that having social support locally in Hong Kong is much more important for a worker’s mental health than physical health, with an MCS estimate nine times of the PCS counterpart obtained with Method 6; presumably such support can have immediate effect on her mental wellbeing. The lone surprising finding is the negative association between participation in religious activities and one’s mental and physical wellbeing, which goes against what was suggested in the literature (Nakonz and Shik, 2009). A possible explanation is reversed causation: Those with mental and physical problems may want to seek out support in religion more so than those without such problems.

A female migrant domestic worker’s working conditions matter. Compared to those specific working conditions such as long working hours, lack of vacation days, lack of privacy, and improper sleeping space reported in the literature (Hollingsworth, 2017; Huang and Yeoh, 2007; Sum, 2019; Wong, 2010), other factors appear to be more important: Her monthly income is positively related to her mental health strongly and to her physical health modestly. Receiving bonuses and gifts from her employer tends to give a strong boost to her mental as well as physical wellbeing; on the other hand, having had back-pay experiences appears to be negatively related to her wellbeing, especially her mental wellbeing. Also important is her employer’s poor attitudes, which tend to be negatively associated with her mental health. In summary, we found strong evidence for the association between a Filipina migrant domestic worker’s mental/physical health and the social support she received as well as the working conditions she was subject to. The negative association between back-pay experiences and employers’ poor attitudes on the one hand and mental health on the other teased out the general finding of the statistically significant relationship between poor employment conditions and poor mental health established by a recent study using an online 2020–2021 sample of the same population (Sumerlin et al., 2024).

The joint analysis of MCS and PCS allowed us to compare relative effects of various factors when the MCS-PCS correlation is already modeled in the analysis and the two measurement models used the same scaling parameter. This represents a significant step forward in our understanding of the factors associated with these migrant workers’ subjective mental and physical health. In addition to the differential effect of friendship ties in Hong Kong, agency fees, religious activities, and back pay experiences all show a much stronger negative effect—and receiving bonuses/gifts from her employer displays a much greater positive effect—on a worker’s mental health than on her physical health. These findings are sensible because mental health effects tend to be more immediate, and the factors discussed above are likely to have an immediate impact on one’s mental wellbeing. In comparison with Chung and Mak’s (2020) study using the same survey, our joint analysis of mental and physical health revealed an additional advantage: It highlighted the moderate and indirect impact of certain factors on physical health, which is omitted in separate analysis. For instance, while agency fees, religious activities, and back-pay experiences were previously found to have no impact on physical health in their study, our joint analysis showed that these factors do affect physical health when mental health is considered jointly. More importantly, their analysis of separately modeling the PCS and MCS dimensions by using the standard US-based scoring method yielded a positively age effect on PCS (older, better physical health) yet negative age effect on MCS (older, poorer mental health), implying a negative MCS-PCS correlation, going against the consensus of the literature that suggests a positive MCS-PCS correlation. Furthermore, our analysis definitively establishes the joint impact of work conditions in terms of having received bonuses/gifts and having had back-pay experiences on both mental and physical health, beyond the recent single-dimensional study of either mental health only or mental and physical health separately (Chung and Mak, 2020; Sumerlin et al., 2024).

## Conclusion

In this paper, we reported an analysis based on bootstrapped empirical data and another analysis based on the original empirical survey data for comparing the performance of eight scoring methods, with six of which analyzing the data by assuming a bivariate standard normal (or logistic) distribution to estimate the MCS and PCS dimensions using an SF-12 instrument for measuring mental and physical health. Based on these analyses, for properly analyzing data from the SF-12 version 2 instrument obtained from non-US societies, we strongly recommend the use of the latent confirmatory factor analysis model (Method 6) that assumes a normal distribution and an identity link function for scoring the MCS and PCS dimensions simultaneously. For researchers without access to software for estimating bivariate latent variable models, the use of the basic summary index may suffice. However, cautions must be exercised because, as the bootstrapped analysis indicated, such estimates may sometimes fall outside of reasonable range. Our empirical analysis of the original Filipina migrant workers’ sample also supports the choice of Method 6.

This paper has made two significant contributions to the methodological and the substantive literatures: It represents a first attempt at evaluating the relative performance of eight scoring methods with both a bootstrapped and a non-bootstrapped analysis using the data from the Filipina migrant domestic workers surveyed in Hong Kong in 2017 to definitively determine the most appropriate scoring method (i.e., the latent confirmatory factor analysis model that assumes a normal distribution and an identity link function for measuring the MCS and PCS dimensions jointly). Furthermore, our study also represents the first empirical analysis of these female migrant workers’ subjective mental and physical health jointly when the MCS-PCS association is taken into consideration together, thereby enabling direct comparability of estimated MCS and PCS effects on the same scale for drawing substantive conclusions that go beyond the past and recent research relying on separate MCS and PCS analyses.

## Data Availability

The data analyzed in this study is subject to the following licenses/restrictions: the data are currently not publicly available but can be provided upon a written request. Requests to access these datasets should be directed to tfliao@illinois.edu.

## Appendix A: Specification of the Eight Scoring Methods

In all the specifications of the SF-12 v. 2 item below, *bp*=bodily pain, *gh*=general health, *mh*=mental health, *ph*=physical function, *re*=role-emotional, *rp*=role-physical, *sf*=social functioning, and *vt*=vitality, respectively. Both *bp* and *mh* are reverse coded before computation.

Method 1:

$$MCS=50+10\left(\begin{array}{l} -0.09731BP-0.01571GH+0.48581MH\\ -0.22999PF+0.43407RE-0.12329RP\\ +0.26876SF+0.23534VT \end{array}\;\right)$$

$$PCS=50+10\left(\begin{array}{l} 0.31754BP+0.24954GH-0.22069MH+0.42402PF\\ -0.19206RE+0.35119RP-0.00753SF+0.02877VT \end{array}\right)$$

where all capitalized variables are standardized, with the twin items of *mh*, *pf*, *re*, and *rp* combined first, respectively.

Method 2:

$$MCS=\frac{1}{6}\left(mh1+mh2+re1+re2+sf+vt\right)$$

$$PCS=\frac{1}{6}\left(\mathrm{bp}+gh+\frac{5}{3}pf1+\frac{5}{3}pf2+rp1+rp2\right)$$

Methods 3:

$$g\left(y_{j}\right)=\alpha_{j}+\beta_{j}MCS+\varepsilon_{j}$$

where *y_j_*=*mh*1, *mh*2, *re*1, *re*2, *sf*, or *vt*, and *g*(·) is the ordinal logit link function assuming the logistic distribution

$$g\left(y_{j}\right)=\alpha_{jk}+\beta_{j}PCS+\varepsilon_{j}$$

where *y_j_*=*bp*, *gh*, *pf*1, *pf*2, *rp*1, or *rp*2, and *g*(·) is the ordinal logit link function assuming the logistic distribution, and

$$cov\left(MCS,PCS\right)\neq 0$$

Methods 4:

$$g\left(y_{j}\right)=\alpha_{j}+\beta_{j}MCS+\varepsilon_{j}$$

where *y_j_*=*mh*1, *mh*2, *re*1, *re*2, *sf*, or *vt*, and *g*(·) is the identity link function assuming the normal distribution

$$g\left(y_{j}\right)=\alpha_{j}+\beta_{j}PCS+\varepsilon_{j}$$

where *y_j_*=*bp*, *gh*, *pf*1, *pf*2, *rp*1, or *rp*2, and *g*(·) is the identity link function assuming the normal distribution, and

$$cov\left(MCS,PCS\right)\neq 0$$

Methods 5:

$$g\left(y_{j}\right)=\alpha_{j}+\beta_{j}MCS+\varepsilon_{j}$$

where *y_j_*=*gh*, *mh*1, *mh*2, *re*1, *re*2, *sf*, or *vt*, and *g*(·) is the ordinal logit link function assuming the logistic distribution with cross-loaded *gh* and *vt* subscales

$$g\left(y_{j}\right)=\alpha_{j}+\beta_{j}PCS+\varepsilon_{j}$$

where *y_j_*=*bp*, *gh*, *pf*1, *pf*2, *rp*1, *rp*2, or *vt*, and *g*(·) is the ordinal logit link function assuming the logistic distribution, and

$$cov\left(MCS,PCS\right)\neq 0$$

Methods 6:

$$g\left(y_{j}\right)=\alpha_{j}+\beta_{j}MCS+\varepsilon_{j}$$

where *y_j_*=*gh*, *mh*1, *mh*2, *re*1, *re*2, *sf*, or *vt*, and *g*(·) is the identity link function assuming the normal distribution with cross-loaded *gh* and *vt* subscales

$$g\left(y_{j}\right)=\alpha_{j}+\beta_{j}PCS+\varepsilon_{j}$$

where *y_j_*=*bp*, *gh*, *pf*1, *pf*2, *rp*1, *rp*2, or *vt*, and *g*(·) is the identity link function assuming the normal distribution, and

$$cov\left(MCS,PCS\right)\neq 0$$

Methods 7:

$$g\left(y_{j}\right)=\alpha_{j}+\beta_{j}MCS+\varepsilon_{j}$$

where *y_j_*=*mh*1, *mh*2, *re*1, *re*2, *sf*, or *vt*, *g*(·) is the identity link function assuming the normal distribution, cov(*ε*_*mh*1_, *ε*_*mh*2_)≠0, and cov(*ε*_*re*1_, *ε*_*re*2_)≠0;

$$g\left(y_{j}\right)=\alpha_{j}+\beta_{j}PCS+\varepsilon_{j}$$

where *y_j_*=*bp*, *gh*, *pf*1, *pf*2, *rp*1, or *rp*2, and *g*(·) is the identity link function assuming the normal distribution, cov(*ε*_*pf*1_, *ε*_*pf*2_)≠0, and cov(*ε*_*rp*1_, *ε*_*rp*2_)≠0; and

$$cov\left(MCS,PCS\right)\neq 0$$

Methods 8:

$$g\left(y_{j}\right)=\alpha_{j}+\beta_{j}MCS+\varepsilon_{j}$$

where *y_j_*=*gh*, *mh*1, *mh*2, *re*1, *re*2, *sf*, or *vt*, *g*(·) is the identity link function assuming the normal distribution with cross-loaded *gh* and *vt* subscales, cov(*ε*_*mh*1_,*ε*_*mh*2_)≠0, and cov(*ε*_*re*1_*ε*,_*re*2_)≠0;

$$g\left(y_{j}\right)=\alpha_{j}+\beta_{j}PCS+\varepsilon_{j}$$

where *y_j_*=*bp*, *gh*, *pf*1, *pf*2, *rp*1, *rp*2, or *vt*, and *g*(·) is the identity link function assuming the normal distribution, cov(*ε*_*pf*1_, *ε*_*pf*2_)≠0, and cov(*ε*_*rp*1_,*ε*_*rp*2_)≠0; and
