# Supplementary material for: A study of Filipina migrant workers’ subjective health in Hong Kong and an assessment of eight scoring methods for the 12-Item Short Form Health Survey (SF-12)
Source: Front Sociol. 2025 Jan 15;9:1420017. doi: 10.3389/fsoc.2024.1420017 (PMC11776024; doi:10.3389/fsoc.2024.1420017)
Supplement: Supplementary file 1 [file Data_Sheet_1.docx]

**Appendix A**: Specification of the Eight Scoring Methods

In all the specifications of the SF-12 v. 2 item below, *bp*=bodily pain, *gh*=general health, *mh*=mental health, *ph*=physical function, *re*=role-emotional, *rp*=role-physical, *sf*=social functioning, and *vt*=vitality, respectively. Both *bp* and *mh* are reverse coded before computation.

**Method 1**:

$$MCS=50+10\left（ -0.09731BP-0.01571GH+0.48581MH-0.22999PF+0.43407RE-0.12329RP+0.26876SF+0.23534VT \right）$$

$$PCS=50+10\left（ 0.31754BP+0.24954GH-0.22069MH+0.42402PF-0.19206RE+0.35119RP-0.00753SF+0.02877VT \right）$$

where all capitalized variables are standardized, with the twin items of *mh*, *pf*, *re*, and *rp* combined first, respectively.

**Method 2**:

$$MCS=\frac{1}{6}\left( mh1+mh2+re1+re2+sf+vt \right)$$

$$PCS=\frac{1}{6}\left( bp+gh+\frac{5}{3}pf1+\frac{5}{3}pf2+rp1+rp2 \right)$$

**Methods 3**:

$$g\left( y_{j} \right)=\alpha_{j}+\beta_{j}MCS+\varepsilon_{j}$$

where *y_j_*=*mh*1, *mh*2, *re*1, *re*2, *sf*, or *vt*, and *g*(·) is the ordinal logit link function assuming the logistic distribution

$$g\left( y_{j} \right)=\alpha_{jk}+\beta_{j}PCS+\varepsilon_{j}$$

where *y_j_*=*bp*, *gh*, *pf*1, *pf*2, *rp*1, or *rp*2, and *g*(·) is the ordinal logit link function assuming the logistic distribution, and

$$cov\left( MCS,PCS \right)\neq0$$

**Methods 4**:

$$g\left( y_{j} \right)=\alpha_{j}+\beta_{j}MCS+\varepsilon_{j}$$

where *y_j_*=*mh*1, *mh*2, *re*1, *re*2, *sf*, or *vt*, and *g*(·) is the identity link function assuming the normal distribution

$$g\left( y_{j} \right)=\alpha_{j}+\beta_{j}PCS+\varepsilon_{j}$$

where *y_j_*=*bp*, *gh*, *pf*1, *pf*2, *rp*1, or *rp*2, and *g*(·) is the identity link function assuming the normal distribution, and

$$cov\left( MCS,PCS \right)\neq0$$

**Methods 5**:

$$g\left( y_{j} \right)=\alpha_{j}+\beta_{j}MCS+\varepsilon_{j}$$

where *y_j_*=*gh*, *mh*1, *mh*2, *re*1, *re*2, *sf*, or *vt*, and *g*(·) is the ordinal logit link function assuming the logistic distribution with cross-loaded *gh* and *vt* subscales

$$g\left( y_{j} \right)=\alpha_{j}+\beta_{j}PCS+\varepsilon_{j}$$

where *y_j_*=*bp*, *gh*, *pf*1, *pf*2, *rp*1, *rp*2, or *vt*, and *g*(·) is the ordinal logit link function assuming the logistic distribution, and

$$cov\left( MCS,PCS \right)\neq0$$

**Methods 6**:

$$g\left( y_{j} \right)=\alpha_{j}+\beta_{j}MCS+\varepsilon_{j}$$

where *y_j_*=*gh*, *mh*1, *mh*2, *re*1, *re*2, *sf*, or *vt*, and *g*(·) is the identity link function assuming the normal distribution with cross-loaded *gh* and *vt* subscales

$$g\left( y_{j} \right)=\alpha_{j}+\beta_{j}PCS+\varepsilon_{j}$$

where *y_j_*=*bp*, *gh*, *pf*1, *pf*2, *rp*1, *rp*2, or *vt*, and *g*(·) is the identity link function assuming the normal distribution, and

$$cov\left( MCS,PCS \right)\neq0$$

**Methods 7**:

$$g\left( y_{j} \right)=\alpha_{j}+\beta_{j}MCS+\varepsilon_{j}$$

where *y_j_*=*mh*1, *mh*2, *re*1, *re*2, *sf*, or *vt*, *g*(·) is the identity link function assuming the normal distribution, cov($\varepsilon$*_mh_*_1_*,*$\varepsilon$*_mh_*_2_)≠0, and cov($\varepsilon$*_re_*_1_*,*$\varepsilon$*_re_*_2_)≠0;

$$g\left( y_{j} \right)=\alpha_{j}+\beta_{j}PCS+\varepsilon_{j}$$

where *y_j_*=*bp*, *gh*, *pf*1, *pf*2, *rp*1, or *rp*2, and *g*(·) is the identity link function assuming the normal distribution, cov($\varepsilon$*_pf_*_1_*,*$\varepsilon$*_pf_*_2_)≠0, and cov($\varepsilon$*_rp_*_1_*,*$\varepsilon$*_rp_*_2_)≠0; and

$$cov\left( MCS,PCS \right)\neq0$$

**Methods 8**:

$$g\left( y_{j} \right)=\alpha_{j}+\beta_{j}MCS+\varepsilon_{j}$$

where *y_j_*=*gh*, *mh*1, *mh*2, *re*1, *re*2, *sf*, or *vt*, *g*(·) is the identity link function assuming the normal distribution with cross-loaded *gh* and *vt* subscales, cov($\varepsilon$*_mh_*_1_*,*$\varepsilon$*_mh_*_2_)≠0, and cov($\varepsilon$*_re_*_1_*,*$\varepsilon$*_re_*_2_)≠0;

$$g\left( y_{j} \right)=\alpha_{j}+\beta_{j}PCS+\varepsilon_{j}$$

where *y_j_*=*bp*, *gh*, *pf*1, *pf*2, *rp*1, *rp*2, or *vt*, and *g*(·) is the identity link function assuming the normal distribution, cov($\varepsilon$*_pf_*_1_*,*$\varepsilon$*_pf_*_2_)≠0, and cov($\varepsilon$*_rp_*_1_*,*$\varepsilon$*_rp_*_2_)≠0; and
